# Can Pregnancy Experience Predict Birth Experience, Postpartum Depression and Anxiety? A Prospective Descriptive Study

**DOI:** 10.1002/nop2.70116

**Published:** 2024-12-06

**Authors:** Monireh Moniri, Mojgan Mirghafourvand, Shahla Meedya, Solmaz Ghanbari‐Homaie

**Affiliations:** ^1^ Students Research Committee Tabriz University of Medical Sciences Tabriz Iran; ^2^ Social Determinants of Health Research Center Tabriz University of Medical Sciences Tabriz Iran; ^3^ Western Sydney University Sydney Australia; ^4^ Assistant Professor, Department of Midwifery, Faculty of Nursing and Midwifery Tabriz University of Medical Sciences Tabriz Iran

**Keywords:** birth satisfaction, depression, Iran, postpartum anxiety, pregnancy stress

## Abstract

**Aim:**

A positive pregnancy experience can be a good start for healthy motherhood. This study aimed to investigate Iranian women's pregnancy experience and how self‐reported hassles and uplifts influence birth experience, postpartum depression and anxiety, and the association between childbirth experience and postpartum mental health.

**Design:**

A prospective descriptive study.

**Methods:**

A prospective descriptive study was conducted among 228 pregnant women from health centres in Tabriz, Iran. From the 28th to 36th weeks of pregnancy, participants completed the Pregnancy Experience Scale. Then, the mothers were followed up until 4–6 weeks postpartum, and Childbirth Experience Questionnaires version 2.0, Edinburgh Postnatal Depression and the short form of Specific Postpartum Anxiety Scales were completed. The data were analysed using the general linear model.

**Results:**

After adjusting for possible confounding variables, there was no statistically significant association between women's pregnancy and childbirth experiences. However, the mean scores of postpartum depression and anxiety were significantly higher in women who felt unhappy about the discomforts that they experienced during pregnancy (*β* [95% CI] = 0.01 [0.01–0.02]; *p* < 0.001, 0.22 [0.09–0.35]; *p* = 0.001, respectively).

**Conclusion:**

There was a significant statistical reverse association between childbirth experience and postpartum anxiety and depression. The study demonstrated a significant association between women's pregnancy, birth experiences and postpartum psychological outcomes. Implementing interventions that create a positive pregnancy experience will likely have an impact on reducing the prevalence of postpartum depression and anxiety.

**Patient or Public Contribution:**

Pregnant women participated solely in the data collection by responding to the questionnaires. No participant contributions were required for the study's design, outcome measurement or implementation.

## 1 Introduction

Pregnancy is a unique experience for women. During this period, physiological processes occur in a woman's body, which is associated with significant emotional and psychological changes for women (Vehmeijer et al. 2019). The World Health Organization (WHO) recommends providing care so that ‘every pregnant woman and newborn receives quality care throughout the pregnancy, childbirth, and postnatal period’. Prenatal quality care based on human rights does not simply mean reducing mortality and disease burden. Instead, prenatal care should be designed and implemented in a dignified and respectful way that ensures a satisfactory experience for women (WHO 2016). A positive experience for women during pregnancy is defined as maintaining the natural physical and cultural‐social condition where a woman's self‐esteem, sense of competence and independence are strengthened (Downe et al. 2016).

Although pregnancy is considered a positive event, women's mind–body interactions during pregnancy and childbirth can affect their mental health and physical conditions (Anderson 2021). For example, most pregnant women feel pleased when they feel their babies' movements or hear their heartbeats. Some women have a spiritual sense about being pregnant and consider being treated special by others as an uplifting experience (Sanaeinasab et al. 2021). On the other hand, women are concerned for various reasons, such as parenting ability, the child's well‐being, returning to work and social or interpersonal matters (Goldfinger et al. 2020). Physical conditions such as common pregnancy symptoms and pregnancy hassles may influence their mental health and vice versa (Gooley, Mohapatra, and Twan 2018; Harmel and Höfelmann 2022).

The increase in the mother's anxiety level during pregnancy is associated with adverse consequences, such as postpartum depression (Grigoriadis et al. 2019), negative childbirth experience, poor mental health, perceived bonding impairment (Azimi, Fahami, and Mohamadirizi 2018; Nath et al. 2019), a reluctance to start breastfeeding (Horsley et al. 2019) and unfavourable growth and development of the newborn (Graham et al. 2020). Furthermore, emotional and psychological problems experienced during pregnancy can increase the risk of postpartum physical complications. For example, worry and stress have a negative effect on the health of the mother and foetus during pregnancy and after birth (Araji et al. 2020; Van Den Heuvel et al. 2021).

### 1.1 Background

Although positive and negative pregnancy experiences can have consequences for the mother and child, few studies have been done regarding this topic. A study in the United States has been done to investigate whether severe anxiety during pregnancy can predict postpartum depression or not. According to the results of this study, excessive worry was a strong predictor of postpartum depressive symptoms (Osborne et al. 2021).

A longitudinal study investigated the impact of prenatal emotional problems on postpartum stress among 51 women in Romania. Based on the results of this study, pregnancy problems such as anxiety and negative experiences were predictors of maternal postpartum stress and the quality of mother–child interaction (Paica 2019). Studies show that pregnancy complications like gestational hypertension can worsen depression, anxiety and post‐traumatic stress disorder (PTSD) symptoms. This correlation may be related to hypertension and its unfavourable effects, such as premature birth or prenatal events (Roberts, Davis, and Homer 2019). Another systematic review found the global prevalence of postpartum depression to be 14% (5%–26.3%, depending on the nation). This study found that gestational diabetes, a male foetus, a history of depression during pregnancy and epidural anaesthesia during childbirth were risk factors for postpartum depression. (Liu, Wang, and Wang 2022). A systematic review study found that life events, relationship quality, partner and mother support and chronic strain are important predictors of postpartum depression (Yim et al. 2015).

To the best of our knowledge, no study has assessed the relationship between pregnancy and childbirth experiences. According to a review study, the prevalence of negative childbirth experiences ranges from 6.8% to 44%. Predictors of a negative childbirth experience include individual factors (age, parity, fear, self‐efficacy, participation, control, expectations and preparation); interpersonal relationships (spouse support and caregiver support); and unexpected medical issues (prolonged labour, induction and augmentation, forceps delivery, emergency caesarean section, use of analgesics in the mother, low Apgar score and neonatal transfer to intensive care) (Hosseini‐Tabaghdehi et al. 2020).

The childbirth experience may be associated with postpartum outcomes. A Norwegian cohort study found that negative childbirth experience, depression history and pre‐pregnancy pain predicted postpartum depression (Rosseland et al. 2020). A UK study indicated that postpartum maternal anxiety was higher among women with high mental stress and negative emotional responses to labour and birth (Fallon et al. 2022).

Many studies have explored the association between pregnancy psychological factors and postpartum outcomes. For instance, many studies investigated the association between pregnancy anxiety, depression, or mental health and postpartum anxiety, depression, PTSD and other postpartum psychological outcomes. However, there are limited studies on the relationship between pregnancy experience (uplifts and hassles) with specific and standard scales and postpartum psychological outcomes. Also, some studies considered anxiety, distress or depression during pregnancy as a pregnancy experience. However, the pregnancy experience is multifaceted and should be examined in its entirety. On the other hand, these consequences have not been studied in Iran. Therefore, it is essential to determine the association of pregnancy experience with postpartum outcomes, which can be effective in designing evidence‐based interventions to improve postpartum outcomes.

### 1.2 Aim

This study aimed to investigate Iranian women's pregnancy experience and how self‐reported hassles and uplifts influence childbirth experience, postpartum depression and anxiety, as well as the association between childbirth experience and postpartum mental health.

## 2 Methods

### 2.1 Study Design

This is a prospective descriptive study to determine Iranian women's pregnancy experience and how the self‐reported hassles and uplifts influence childbirth experience, postpartum depression and anxiety, as well as the association between childbirth experience and postpartum depression and anxiety. A total of 228 pregnant women were included in the study between January 2022 and February 2023. Overall, four women were lost to follow‐up during the postpartum period.

### 2.2 Eligibility Criteria

Inclusion criteria included living in Tabriz city (to access and follow mothers after birth); a gestational age of 28–36 weeks at the time of entry into the study (before 28 weeks, there is a possibility of miscarriage or foetal loss, and after 36 weeks, there is a short period until childbirth, which can affect women's feelings and concerns and cause bias in reporting their pregnancy experiences); and singleton pregnancy (multiple pregnancy has special complications). Exclusion criteria included having more than three childbirths (these women usually have a more complicated pregnancy and birth period); a history of depression or postpartum depression (these women are more likely to have postpartum depression); taking antidepressants; having other mental health disorders; divorce during pregnancy; having a known abnormality in the foetus; and the occurrence of a stressful life event in the last 3 months as reported by the mother (death or incurable disease diagnosis in a first‐degree family member).

### 2.3 Sampling Process

Sampling was done using the random cluster method. First, a quarter of all the health centres in Tabriz, Iran (82 centres) were randomly selected. A list of all women with a gestational age of 28–36 weeks was extracted from the electronic health site of each centre. Then, women were randomly selected from the prepared list. Using the phone number registered in each file, the researcher made a phone call with the chosen women and, while briefly explaining the study's objectives, examined them regarding eligibility criteria. Women eligible and willing to participate in the study were asked to specify a time for a face‐to‐face visit. After obtaining written informed consent, a demographic checklist and Pregnancy Experience Questionnaire–Brief Version (PES) were completed by interview during the face‐to‐face meeting. Then, the mothers were followed up until 4–6 weeks after birth, and the Childbirth Experience Questionnaires Version 2.0 (CEQ 2.0), Edinburgh Depression Questionnaire (EPDS) and the Short Form of the Postpartum Anxiety Questionnaire (PSAS‐RSF) were completed. Questionnaires were mainly completed through face‐to‐face interviews (60%); however, women who were reluctant to present in the health centre (due to the COVID‐19 pandemic) completed the questionnaires either through phone interviews (35%) or electronic links provided in social networks (5%).

### 2.4 Study Setting

The study included participants from urban health centres in Tabriz (East Azerbaijan, Iran). Tabriz has a population of about 1.5 million and is the third largest city in Iran. Most health centres are located near women's places of residence, and their demographic and health information is recorded electronically in those centres.

### 2.5 Data Collection Tool

Four main scales were used in this study: the Pregnancy Experience Scale–Brief Version (PES), the Childbirth Experience Questionnaire 2.0 (CEQ 2.0) and the Edinburgh Postnatal Depression and Postpartum‐Specific Anxiety Scale‐Research Short‐Form (PSAS‐RSF). Demographic and obstetrics data were obtained through researcher‐made questionnaires.

### 2.6 Demographic and Obstetrics Checklist

The demographic and obstetric checklist included questions about age, income, education, job, Body Mass Index (BMI), gestational age, parity, history of abortions, stillbirth, wanted pregnancy, planned pregnancy, prenatal complications and the type of birth. Weight and height were extracted from the woman's electronic health record and inserted into the BMI formula (weight [kg]/height^2^ [m]) and BMI calculated.

### 2.7 Pregnancy Experience Scale–Brief Version (PES)

This questionnaire consists of 20 questions, with two subscales: uplifts and hassles. Ten questions assess women's feelings of happiness, for example, ‘spiritual feelings about being pregnant’, ‘thinking about the baby's appearance’ and ‘receiving assistance because of pregnancy’ (uplifts domain). The other 10 questions evaluate the feelings of worry during pregnancy, for example, ‘body changes due to pregnancy’, ‘concerns about physical symptoms’ and ‘thoughts about whether the baby is normal’ (hassles domain). Each question is graded on a 4‐point Likert scale: not at all (score 0), little (score 1), a lot (score 2) and very much (score 3). A higher score indicates more worry or happiness (DiPietro et al. 2004; DiPietro, Christensen, and Costigan 2008). The Persian version of the scale has been psychometrically evaluated by Ebadi et al. (Cronbach's *α*= 0.71) (Ebadi, Kariman, and Hajifoghaha 2017).

### 2.8 Childbirth Experience Questionnaire 2.0 (CEQ 2.0)

The revised version of the questionnaire contains 23 questions with four subscales (own capacity, professional support, perceived safety and participation). There are 20 questions with four options (completely agree [score 4], often agree [score 3], often disagree [score 2] and completely disagree [score 1]) and three questions in the form of a visual scale from 0 to 100. These scores are converted into values between 1 and 4: 0–40 (score 1), 41–60 (score 2), 61–80 (score 3) and 81–100 (score 4). Negative questions (3, 5, 8, 13, 14, 19, 20 and 21) are scored reversely. High average scores in this tool mean a more positive childbirth experience. The Persian version of the scale has been psychometrically evaluated in Iran by Ghanbari et al. (Cronbach's *α*= 0.93) (Dencker et al. 2010; Ghanbari‐Homayi et al. 2019).

### 2.9 Edinburgh Postnatal Depression

This questionnaire contains 10 questions, and the answers are graded on a 4‐point Likert scale of 0–3. The total score of the Edinburgh scale is between 0 and 30, and a score of 12 or more is considered for the risk of postpartum depression. Questions 3, 5, 6, 7, 8, 9 and 10 are scored reversely (Cox, Holden, and Sagovsky 1987). Montazeri, Torkan, and Omidvari (2007) have psychometrically evaluated the Persian version of the scale.

### 2.10 Postpartum‐Specific Anxiety Scale‐Research Short‐Form (PSAS‐RSF)

This questionnaire was designed to determine the frequency (not the intensity) of mothers' anxiety up to 12 months after birth. This questionnaire contains 12 questions and shows the woman's feelings in the last 7 days, including emotional distress and worries about the newborn. Scoring is on a 4‐point Likert scale, and the options include not at all (score 1), sometimes (2), often (3) and almost always (4). A total score of 48 or higher indicates more anxiety (Davies et al. 2021). The Persian version of the scale has been psychometrically evaluated by Hasanzadeh et al. (2021).

### 2.11 Sample Size

The sample size of 152 pregnant women was calculated based on the results of Khalife‐Ghaderi and Amiri‐Farahani's study and considering the standard deviation of 5.87, *α* = 0.05 and *d* = 0.05 around the mean of 18.67 (Khalife‐Ghaderi and Amiri‐Farahani 2021) through the *n* = *Z*_1−a/2_ sd^2^/*d* ^2^ formula. Considering that the sampling type was a cluster, the sample size was multiplied by 1.5 (the design effect) and the final sample size was calculated as 228 women.

### 2.12 Ethical Considerations

This study has been approved by the ethics committee of Tabriz University of Medical Sciences, Tabriz, Iran (IR.TBZMED.REC.1400.909). Written informed consent was obtained from all participants, who were assured that their information would remain confidential. To preserve the confidentiality of the data, the questionnaire's coding method was used without mentioning the name or surname.

### 2.13 Data Analysis

The data results were analysed using SPSS, Version 24.0 for Windows software (IBM Inc., Armonk, NY, USA). The quantitative variables (such as age, gestational age and BMI) with a normal distribution were reported with the mean (standard deviation), and, in the absence of a normal distribution, we reposted with the median (25th–75th percentile). Qualitative variables (education, income satisfaction, planned pregnancy and postpartum complications) were also reported as frequency (percentage). The means were compared with the independent *t*‐test and the one‐way ANOVA test. To determine the association between pregnancy experience and childbirth experience, postpartum depression and anxiety, Pearson's correlation coefficient in univariate analysis and the general linear model in multivariate analysis were used with an adjustment of socio‐demographic and obstetric characteristics (*p* < 0.1 was used as the threshold for including variables in the model) (Bursac et al. 2008). The postpartum depression score did not have a normal distribution, and its log was included in the model. A *p* < 0.05 was considered to be statistically significant.

## 3 Results

A total of 228 pregnant women from a population size of 320 were included in the study between January 2022 and February 2023 (response rate: 71%). Overall, four women were lost to follow‐up during the postpartum period. Among 228 women who participated in the study, the mean age was 29.0 (5.9) years, and the mean gestational age was 31.9 (2.7) weeks. About three quarters of women (73.2%) stated that their pregnancy was wanted, and less than half of the women were primiparous (43.4%) and gave birth vaginally (Table 1).

**TABLE 1:** Socio‐demographic and obstetric characteristics of participants (*N* = 228).

Variables	*n* (%)	Variables	*n* (%)
Age, (years), mean (SD)	29.0 (5.9)	Wanted sex of baby	
Husband age, (years), mean (SD)	33.9 (5.7)	Yes	180 (78.9)
Education		No	48 (21.1)
Under diploma	80 (35.1)	Satisfaction with pervious birth	
Diploma	91 (39.1)	Yes	78 (67.8)
Academic	57 (25.0)	No	37 (32.2)
Husband education		Complications during pregnancy^a^	
Under diploma	86 (37.7)	Yes	53 (23.2)
Diploma	87 (38.2)	No	175 (76.8)
Academic	55 (24.1)	Doula support	
Job		Yes	80 (35.7)
Housewife	215 (94.3)	No	144 (64.3)
Employed	13 (5.7)	Companion during labour	
Husband job		Yes	132 (58.9)
Employed	37 (16.2)	No	92 (41.1)
Worker	48 (21.1)	Birth attendant	
Self‐employed	143 (62.7)	Obstetrician/Obstetrician resident	171 (76.3)
Income satisfaction		Midwifery/midwifery student	53 (23.7)
Not enough	51 (22.4)	Type of birth	
Relatively enough	154 (67.5)	Vaginal	99 (43.4)
Completely enough	23 (10.1)	Emergency caesarean section	55 (24.1)
Gestational age at the time of entering the study (Weeks), Mean (SD)	31.9 (2.7)	Elective caesarean section	74 (32.5)
BMI (kg/m^2^), Mean (SD)	24.9 (4.4)	Place of birth	
Parity		Teaching	63 (28.1)
1	99 (43.4)	Governmental	65 (29.0)
2	84 (36.8)	Private	96 (42.9)
3 or more	45 (19.7)	Postpartum complication^b^	
History of abortion		Yes	29 (12.7)
Yes	41 (18.0)	No	199 (87.3)
No	187 (82.0)	Skin to skin contact	
History of stillbirth		Yes	190 (84.8)
Yes	3 (1.3)	No	34 (15.2)
No	225 (98.7)	Foetal disease	
Wanted pregnancy		Yes	35 (15.4)
Yes	167 (73.2)	No	193 (84.6)
No	61 (26.8)		
Planned pregnancy			
Yes	159 (69.7)		
No	69 (30.3)		

^a^ Pre‐eclampsia, diabetes mellitus.

^b^ Infection, haemorrhage and thrombosis.

The median (25th, 75th percentile) of postpartum depression was 7 (3, 12). Also, the mean (standard deviation) of anxiety, childbirth experience, uplift subscale and hassles of pregnancy experience was 22.3 (4.7), 2.8 (0.4), 22.2 (4.5) and 1.11 (5.2), respectively (Table 2).

**TABLE 2:** Relationship of pregnancy experience with postpartum depression, childbirth experience and postpartum‐specific anxiety (*N* = 224).

Variables	Mean (Standard deviation)	Median (percentile 25, 75)	Uplifts	Hassles	Childbirth experience
			*r* (*p*‐value)	*r* (*p*‐value)	*r* (*p*‐value)
Postpartum depression (0–30)	8.1 (6.4)	7 (3, 12)	−0.06 (0.327)^a^	0.38 (< 0.001)^a^	−0.31 (< 0.001)^a^
Postpartum‐specific anxiety (12–48)	22.3 (4.7)	22 (18, 25)	0.11 (0.083)^b^	0.25 (< 0.001)^b^	−0.18 (0.007)^b^
Childbirth experience (1–4)	2.8 (0.4)	2.9 (2.7, 3.1)	0.18 (0.027)^b^	−0.16 (0.041)^b^	—
Pregnancy experience			—	—	—
uplifts (0–30)	22. 2 (4.5)	23 (19, 26)	—	—	—
Hassles (0–30)	11.1 (5.2)	10 (8, 15)	—	—	—

^a^ Spearman correlation test.

^b^ Pearson's correlation test.

### 3.1 Pregnancy Experience and Socio‐Demographic and Maternal Factors

We compared women's childbirth experience, postpartum depression and anxiety scores based on their demographic and maternal characteristics (Table 3). Women with high education and low income reported higher postpartum anxiety scores (*p* < 0.05). In contrast, women who had a companion during labour and birth experienced vaginal birth, had fewer postnatal complications, had skin‐to‐skin contact with their babies and had a birth in private hospitals reported low postnatal anxiety scores (*p* < 0.05). Based on Spearman's correlation test results, there was a weak but statistically significant positive correlation between the uplift subscale and the childbirth experience scale (*r* = 0.18; *p* = 0.027). However, the pregnancy hassles subscale negatively correlated with childbirth experience scale scores (*r* = −0.16; *p* = 0.041) (Table 3).

**TABLE 3:** Relationship of socio‐demographic and obstetric characteristics with postpartum depression, childbirth experience and postpartum‐specific anxiety (*N* = 224).

Variable	Postpartum depression		Childbirth experience		Postpartum anxiety	
	Mean (SD)	*p*	Mean (SD)	*p*	Mean (SD)	*p*
Age (years), mean (SD)	0.01*	0.823**	0.06*	0.434**	−0.00*	0.920**
Husband age (years), mean (SD)	0.02*	0.758**	0.06*	0.404**	0.05*	0.377**
Education		0.747***		0.422***		**0.012** ***
Under diploma	8.3 (6.3)		2.7 (0.4)		21.1 (4.0)	
Diploma	8.2 (6.6)		2.8 (0.3)		22.7 (4.7)	
Academic	7.5 (6.5)		2.8 (0.5)		23.5 (5.4)	
Husband education		0.356***		0.338***		**0.001** ***
Under diploma	8.6 (7.1)		2.7 (0.4)		21.2 (3.9)	
Diploma	8.2 (6.7)		2.8 (0.4)		22.2 (4.8)	
Academic	7.0 (4.8)		2.8 (0.3)		24.2 (5.3)	
Job		0.970^****^		0.288***		0.850^****^
Housewife	8.1 (6.5)		2.8 (0.4)		22.3 (4.7)	
Employed	8.0 (5.6)		2.9 (0.3)		22.6 (4.8)	
Husband job		0.327***		0.210***		**0.036** ***
Employed	9.1 (7.4)		2.6 (0.5)		24.1 (5.9)	
Worker	8.8 (6.7)		2.8 (0.3)		21.7 (4.6)	
Self‐employed	7.6 (6.1)		2.8 (0.4)		22.1 (4.4)	
Income satisfaction		0.199***		**< 0.001** ***		**< 0.001** ***
Not enough	9.5 (6.5)		2.5 (0.5)		24.6 (3.7)	
Relatively enough	7.8 (6.4)		2.8 (0.3)		21.9 (4.9)	
Completely enough	7.2 (6.6)		2.9 (0.3)		20.4 (3.9)	
Gestational age (Weeks), Mean (SD)	−0.02*	0.735**	−0.02*	0.745**	−0.31*	**< 0.001** **
BMI (kg/m^2^), Mean (SD)	0.04*	0.499**	0.12*	0.119**	−0.02*	0.710**
Parity		0.368***		0.927***		0.937***
1	7.6 (6.7)		2.8 (0.4)		22.4 (4.7)	
2	8.9 (6.4)		2.8 (0.4)		22.4 (4.7)	
3 or more	7.6 (6.0)		2.78 (0.3)		22.1 (5.0)	
History of abortion		0.498^****^		0.413^****^		0.961^****^
Yes	8.0 (6.9)		2.7 (0.4)		22.8 (5.6)	
No	8.1 (6.4)		2.8 (0.4)		22.2 (4.5)	
History of stillbirth		0.607^****^		0.799^****^		0.195^****^
Yes	10.5 (0.7)		2.9 (0.1)		18.0 (0.0)	
No	8.1 (6.5)		2.8 (0.4)		22.4 (4.7)	
Wanted pregnancy		0.171^****^		0.122^****^		0.625^****^
Yes	7.7 (6.4)		2.8 (0.4)		22.4 (4.8)	
No	9.1 (6.5)		2.7 (0.4)		22.1 (4.6)	
Planned pregnancy		0.186^****^		0.175^****^		0.493^****^
Yes	7.7 (6.5)		2.8 (0.4)		22.5 (4.9)	
No	9.0 (6.3)		2.7 (0.4)		22.0 (4.5)	
Wanted sex of baby		0.077^****^		**0.038** ^****^		0.223^****^
Yes	7.8 (6.8)		2.8 (0.4)		22.1 (4.8)	
No	9.3 (4.5)		2.6 (0.3)		23.1 (4.6)	
Satisfaction with pervious birth		0.883^****^		0.232^****^		0.076^****^
Yes	7.8 (5.8)		2.8 (0.3)		21.3 (4.4)	
No	8.0 (5.8)		2.7 (0.3)		22.8 (4.1)	
Complications during pregnancy^a^		**0.006** ^****^		0.278^****^		0.178^****^
Yes	10.3 (6.4)		2.7 (0.4)		23.1 (4.5)	
No	7.4 (6.3)		2.8 (0.3)		22.1 (4.8)	
Doula support		0.526^****^		0.943^****^		**< 0.001** ^****^
Yes	7.8 (4.1)		2.8 (0.3)		24.5 (4.5)	
No	8.3 (7.4)		2.8 (0.4)		21.1 (4.4)	
Companion during labour		0.350^****^		**0.010** ^****^		**0.038** ^****^
Yes	7.7 (5.3)		2.9 (0.3)		21.5 (4.6)	
No	8.6 (7.8)		2.7 (0.4)		22.9 (4.8)	
Birth attendant		0.339^****^		**0.002** ^****^		**0.004** ^****^
Obstetrician/Obstetrician resident	8.3 (6.6)		2.7 (0.4)		22.8 (4.9)	
Midwifery/midwifery student	7.3 (5.8)		2.9 (0.2)		20.7 (4.0)	
Type of birth		0.599***		0.076***		**< 0.001** ***
Vaginal	7.6 (6.1)		2.8 (0.3)		20.8 (4.0)	
Emergency caesarean section	8.3 (7.1)		2.7 (0.4)		22.8 (4.3)	
Elective caesarean section	8.6 (6.5)				24.1 (5.3)	
Place of birth		0.783***		**0.028** ***		**0.028** ***
Teaching	8.3 (7.0)		2.7 (0.4)		22.4 (5.1)	
Organisational	8.4 (6.6)		2.8 (0.4)		21.1 (4.0)	
Private	7.8 (6.0)		2.9 (0.2)		23.1 (4.9)	
Postpartum complication^b^		0.081^****^		**0.001** ^****^		**0.049** ^****^
Yes	10.1 (6.6)		2.4 (0.4)		24.0 (5.4)	
No	7.8 (6.4)		2.8 (0.3)		22.1 (4.6)	
Skin to skin contact		0.066***		**0.012** ***		**0.037** ***
Yes	7.7 (6.0)		2.8 (0.3)		22.6 (4.8)	
No	10.4 (8.0)		2.6 (0.5)		20.8 (4.2)	

*Note:* Significance for bold values indicates at *p* < 0.05.

^a^ Preeclampsia, Diabetes mellitus.

^b^ Infection, Hemorrhage, Thrombosis.

* *r*.

** Pearson correlation test.

*** One‐way ANOVA test.

^****^ Independent *t*‐test.

### 3.2 Pregnancy Experience and Postpartum Depression

There was a medium and significant statistical association between the pregnancy hassles subscale and Edinburgh postnatal depression scale scores (*r* = 0.38; *p* < 0.001) (Table 2). The mean score of the postpartum depression scale was significantly higher among women who experienced pregnancy hassles (*β* [95% CI] = 0.01 [0.01–0.02]; *p* < 0.001). We could not find an association between the demographic or maternal characteristics of the women and their postpartum depression score (Table 4).

**TABLE 4:** Relationship of pregnancy experience, socio‐demographic and obstetric characteristics with postpartum depression by adjusting of confounder variables (*N* = 224)^a^.

Variables	Postpartum depression	
	B (95% CI)	*p* *
Uplifts	0.00 (−0.00 to 0.01)	0.137
Hassles	0.01 (0.01 to 0.02)	**< 0.001**
Wanted sex of baby (Ref: Yes)		
No	0.03 (−0.08 to 0.14)	0.596
Complications during pregnancy (Ref: Yes)^b^		
No	−0.07 (−0.18 to 0.03)	0.166
Postpartum complication (Ref: Yes)^c^		
No	−0.02 (−0.16 to 0.10)	0.664
Skin to skin contact (Ref: Yes)		
No	0.08 (−0.03 to 0.21)	0.174
*R* ^2^; Adjusted *R* ^2^	0.112; 0.084	

*Note:* Significance for bold values indicates at *p* < 0.05.

^a^ Variables with *p* < 0.1 in univariate analysis (wanted sex of baby, complications during pregnancy, postpartum complication and skin to skin contact) were included in the multivariate analysis.

^b^ Pre‐eclampsia, gestational diabetes.

^c^ Infection, bleeding, thrombosis.

* General linear model test.

### 3.3 Pregnancy Experience and Childbirth Experience

After adjusting for possible confounding variables during labour and birth, there was no statistically significant association between the pregnancy experience scale (hassles and uplifts subscales) and the childbirth experience scale. The mean childbirth experience scale among women who expressed that their pregnancy was unwanted was significantly lower than that of those whose pregnancy was wanted (*β* [95% CI] = −0.17 [−0.32 to −0.02]; *p* = 0.026). Additionally, the mean childbirth experience among women who gave birth in a teaching hospital was statistically significantly lower than that of women who gave birth in a private hospital (*β* [95% CI] = −0.17 [−0.33 to −0.01]; *p* = 0.030). Women who did not experience any complications after childbirth scored higher in childbirth experience compared to women who had complications in the postpartum period (*β* [95% CI] = 0.34 [0.16–0.51]; *p* < 0.001) (Table 5).

**TABLE 5:** Relationship of pregnancy experience, socio‐demographic and obstetric characteristics with childbirth experience by adjusting of confounder variables (*N* = 224)^a^.

Variables	Childbirth experience	
	B (95% CI)	*p* *
Uplifts	0.00 (−0.00 to 0.02)	0.197
Hassles	−0.00 (−0.01 to 0.00)	0.644
Income satisfaction (Ref: Completely enough)		
Not enough	−0.20 (−0.42 to 0.00)	0.053
Relatively enough	0.03 (−0.15 to 0.22)	0.704
Wanted sex of baby (Ref: Yes)		
No	−0.17 (−0.32 to −0.02)	**0.026**
Companion during labour (Ref: Yes)		
No	−0.11 (−0.24 to 0.01)	0.076
Birth attendant (Ref: Midwifery/midwifery student)		
Obstetrician/Obstetrician resident	0.05 (−0.09 to 0.20)	0.490
Type of birth (Ref: Elective caesarean section)		
Vaginal	0.08 (−0.06 to 0.23)	0.261
Emergency caesarean section		
Place of birth (Ref: Private)		
Teaching	−0.17 (−0.33 to −0.01)	**0.030**
Governmental	−0.10 (−0.26 to 0.05)	0.187
Postpartum complication (Ref: Yes)^b^		
No	0.34 (0.16 to 0.51)	**< 0.001**
Skin to skin contact (Ref: Yes)		
No	−0.09 (−0.26 to 0.07)	0.251
*R* ^2^; Adjusted *R* ^2^	0.303; 0.260	

*Note:* Significance for bold values indicates at *p* < 0.05.

^a^ Variables with *p* < 0.1 in univariate analysis (Income satisfaction, wanted sex of baby, companion during labour, birth attendant, type of birth, place of birth, postpartum complication and skin to skin contact) were included in the multivariate analysis.

^b^ Infection, bleeding and thrombosis.

* General linear model test.

### 3.4 Pregnancy Experience and Postpartum Anxiety

The mean score of the postpartum anxiety scale was also significantly higher among women who experienced pregnancy hassles (*β* [95% CI] = 0.22 [0.09–0.35]; *p* = 0.001). There was a significant statistical association between the pregnancy hassles subscale and the postpartum anxiety scale (*r* = 0.25; *p* < 0.001) (Table 2). Women without tertiary education had lower scores on the postpartum anxiety scale compared to women with tertiary education (*β* [95% CI] = −2.64 [−5.12 to −0.16]; *p* = 0.037). However, the mean postpartum anxiety scale score was higher among women with low incomes compared to women with higher incomes (*β* [95% CI] = 3.71 [0.64 to 6.78]; *p* = 0.018) (Table 6).

**TABLE 6:** Relationship of pregnancy experience, socio‐demographic and obstetric characteristics with postpartum anxiety by adjusting of confounder variables (*N* = 224)^a^.

Variables	Postpartum Anxiety	
	B (95% CI)	*p* *
Uplifts	0.12 (−0.03 to 0.29)	0.116
Hassles	0.22 (0.09 to 0.35)	**0.001**
Education (Reference: Academic)		
Under diploma	−0.60 (−3.18 to 1.97)	0.640
Diploma	−0.26 (−2.70 to 2.17)	0.828
Husband education (Ref: Academic)		
Under diploma	−2.64 (−5.12 to −0.16)	**0.037**
Diploma	−2.19 (−4.47 to 0.08)	0.059
Husband job (Ref: Self‐employed)		
Employed	0.29 (−1.50 to 2.08)	0.748
Worker	−2.43 (−5.19 to 0.32)	0.082
Income satisfaction (Ref: Completely enough)		
Not enough	3.71 (0.64 to 6.78)	**0.018**
Relatively enough	0.89 (−1.80 to 3.58)	0.513
Gestational age (Weeks), mean (SD)	−0.19 (−0.45 to 0.07)	0.152
Satisfaction with pervious birth (Ref: Yes)		
No	0.07 (−1.50 to 1.65)	0.925
Doula support (Ref: Yes)		
No	−1.14 (−3.00 to 0.70)	0.221
Companion during labour (Ref: Yes)		
No	−0.29 (−1.99 to 1.40)	0.731
Birth attendant (Ref: Midwifery/midwifery student)		
Obstetrician/Obstetrician resident	−0.96 (−2.90 to 0.97)	0.326
Type of birth (Ref: Elective caesarean section)		
Vaginal	−1.43 (−3.53 to 0.66)	0.178
Emergency caesarean section	−0.51 (−2.90 to 1.88)	0.673
Place of birth (Ref: Private)		
Teaching	0.50 (−1.41 to 2.41)	0.603
Organisational	0.50 (−1.40 to 2.41)	0.600
Postpartum complication (Ref: Yes)^b^		
No	0.21 (−1.96 to 2.39)	0.845
Skin to skin contact (Ref: Yes)		
No	−0.25 (−2.54 to 2.04)	0.829
*R* ^2^; Adjusted *R* ^2^	0.501; 0.384	

*Note:* Significance for bold values indicates at *p* < 0.05.

^a^ Variables with *p* < 0.1 in univariate analysis (Education, husband education, husband job, income satisfaction, gestational age, satisfaction with pervious birth, doula support, companion during labour, birth attendant, type of birth, place of birth, postpartum complication and skin to skin contact) were included in the multivariate analysis.

^b^ Infection, bleeding and thrombosis.

* General linear model test.

### 3.5 Childbirth Experience and Postpartum Depression and Anxiety

**FIGURE 1:**
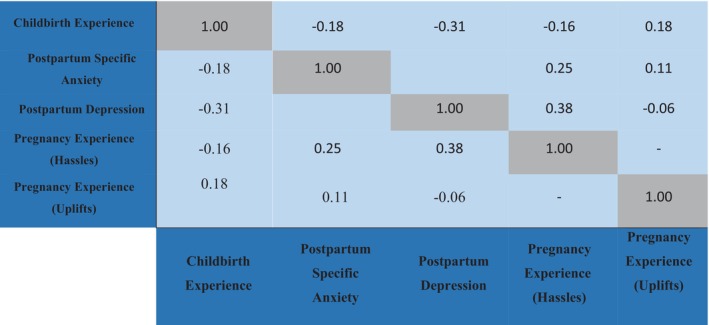
Correlation matrix of pregnancy experience with childbirth experience, postpartum depression and anxiety.

There was a small but significant statistical reverse association between the childbirth experience and postpartum anxiety (*r* = −0.18; *p* = 0.007). Also, there was a medium and significant statistical reverse association between the childbirth experience and postpartum depression (*r* = −0.30; *p* < 0.001) (Table 2, Figure 1).

## 4 Discussion

This study was conducted to determine the association between pregnancy experiences, childbirth experiences, postpartum depression and anxiety levels. The average scores of postpartum depression and anxiety were significantly higher among women who had pregnancy hassles, such as physical symptoms. However, there was no statistically significant association between pregnancy experiences and childbirth experiences.

The results of our study are consistent with those of other studies that focused on pregnancy experiences and postpartum depression and anxiety (Roberts, Davis, and Homer 2019; Obrochta, Chambers, and Bandoli 2020; Osborne et al. 2021; Moulds et al. 2022). The results of a descriptive‐analytical study by Shafaei et al. in Iran showed that there is a statistically significant association between the unpleasant experience of pregnancy and postpartum depression (Sehhatie Shafaei et al. 2008). For instance, the findings of a literature review among 89 studies (Moulds et al. 2022) revealed that repetitive negative thinking, including prenatal worries, was consistently associated with depression and anxiety in late pregnancy and the postpartum period (Roberts, Davis, and Homer 2019). The findings of another literature review showed that mothers who had pregnancy hassles, such as expressing concerns during pregnancy due to complications such as pre‐eclampsia, were more likely to experience postpartum depression and anxiety (Obrochta, Chambers, and Bandoli 2020). A study among 288 Canadian and American women experiencing psychological distress during pregnancy is another example of pregnancy hassle with a great risk for postpartum depression and anxiety (Obrochta, Chambers, and Bandoli 2020). A longitudinal study by Cheng et al. (2021) in Taiwan investigated the trends of stress, anxiety and depression symptoms from pregnancy to postpartum and the prediction of stress and anxiety in postpartum depression. The results of the study showed that the levels of anxiety and depression symptoms increased from the 24th week of pregnancy to postpartum. In contrast, the stress level decreased during pregnancy and increased after birth. More than half of the women had experienced anxiety symptoms, especially at the end of pregnancy and after birth. Women were likely to experience stress, anxiety and depression at the end of pregnancy and postpartum, and anxiety during pregnancy was a predictor of postpartum depression symptoms (Cheng et al. 2021).

The implementation of interventions that create a positive pregnancy experience will likely have an impact on reducing the prevalence of postpartum depression and anxiety as two essential outcomes. For instance, a study in Japan on 32 pregnant women showed that nursing interventions (encouragement of women to record their experiences using a pregnancy diary, recognising and acknowledging negative feelings, reducing the negative aspects of pregnancy, expression and reinforcement of positive feelings, awareness of their own comfortable experiences, promotion of diverse, comfortable aspects and continuation of comfortable experiences) can help to improve the positive experience among pregnant women (Nakamura 2010).

We found no statistically significant association between pregnancy experience and childbirth experience. This study's results are inconsistent with the previous studies (Ghanbari‐Homayi et al. 2019; Khalife‐Ghaderi and Amiri‐Farahani 2021). For instance, in an Iranian study among 225 women, feelings of being uplifted or hassled during pregnancy were one of the predictors of positive or negative childbirth experiences (Khalife‐Ghaderi and Amiri‐Farahani 2021). The reason for the inconsistency could be related to the confounding factors. In our study, we used a general linear regression model where the effect of labour and childbirth variables was adjusted as potential confounding factors. In other studies, the impact of labour and childbirth variables such as type of birth, companion during labour and birth attendant were not considered confounding factors.

The findings of our study in terms of socio‐demographic and maternal factors demonstrated no association between demographic characteristics and postnatal depression; however, those with high education, less income and more pregnancy complications experienced high levels of anxiety. The finding is consistent with the other studies where demographic variables did not significantly correlate with the incidence of postpartum depression (Osborne et al. 2021; van der Zee‐van et al. 2021). However, women with a high maternal education level (Field 2018; van der Zee‐van et al. 2021) and low income reported higher anxiety levels. According to previous studies, people with very low incomes are usually 1.5–3 times more likely to suffer from depression or anxiety compared with people with higher incomes due to their disadvantaged life situations (Ridley et al. 2020).

In our study, women who had an unwanted pregnancy or gave birth in a teaching hospital reported a statistically significantly more negative childbirth experience. Studies have shown that unplanned pregnancy can increase the level of psychological distress in mothers (Muskens et al. 2022). For instance, an Iranian study among 800 primiparous women has reported that the probability of a negative childbirth experience was two times higher among women whose pregnancy was unwanted compared to women who expressed their pregnancy as desired (Ghanbari‐Homayi et al. 2019). Furthermore, giving birth in a teaching hospital was associated with a more negative experience compared to governmental‐based or private hospitals. In teaching hospitals, a multidisciplinary team provides maternity care, including midwives and physicians, without continuity of care models (Ghanbari‐Homayi et al. 2019). Continuity of care during maternity care is well known as an essential factor for a positive birth experience where women feel supported and safe during the care they receive (Alba et al. 2019).

There was a statistically significant association between childbirth experience and postpartum anxiety and depression. Our study's findings are consistent with those of other studies on childbirth experiences and postpartum depression and anxiety. According to the results of a systematic review, 11 out of 15 studies that met the inclusion criteria showed a statistically significant association between childbirth experience and postpartum depression (Bell and Andersson 2016). Despite methodological limitations, evidence suggests that a negative childbirth experience may lead to postpartum depression. A woman giving birth may experience intense fear, helplessness and loss of control (Anderson 2017), thus, her subjective perception of childbirth can impact her psychological well‐being postpartum (Ghanbari‐Homaie et al. 2024).

### 4.1 Strength and Limitations

Using random sampling of the studied participants to prevent bias was one of the strengths of our study. This study also faced some limitations. This study was conducted among pregnant women living in Tabriz, Iran and it may not be generalisable to pregnant women living in rural areas of this city or other parts of the country. As the data collection took place during the COVID‐19 pandemic and we did not measure how the pandemic affected pregnancy, this point can be a limitation of our study. It is possible that the COVID‐19 pandemic and bad experiences of pregnancy and childbirth may increase anxiety and depression in pregnant mothers and negatively affect the results of our study, which were some limitations of this study.

Because women with a history of depression were excluded from the study, the result cannot be generalised to this group of pregnant women. One of the limitations of this study was that the possible impact of the type of data collection method (face‐to‐face, telephone or electronic) on the participants' responses was not analysed. The number of prenatal visits and participation in birth preparation classes was not assessed and evaluated and it could be another limitations of our study.

## 5 Conclusion

The study showed an association between pregnancy experience and postpartum psychological outcomes (depression and anxiety); however, there was no statistically significant association between pregnancy experiences and childbirth experiences. Likely, the implementation of effective and evidence‐based interventions that create a positive pregnancy experience will have an impact on reducing the prevalence of postpartum depression and anxiety as two important postpartum outcomes.

It is suggested that further similar studies especially qualitative studies conducted in other communities where women have different cultural conditions or lifestyles. Designing clinical trials with the aim of evaluating the impact of educational and counselling interventions on the experience of pregnancy, evaluating pregnancy experience and its consequences among high‐risk or adolescent mothers are other suggestions for future studies. Midwives, nurses, medical teams, students and other healthcare workers should receive special training to identify, refer and manage depression, worry and anxiety during pregnancy. To alleviate the psychological burden on women during pregnancy, providing counselling and follow‐up for women after birth could be considered.

## Author Contributions

S.G.‐H., M.M., S.M. and M.M. contributed to the design of the study. M.M. and S.G.‐H. contributed to the implementation and analysis plan. M.M., S.M., M.M. and S.G‐H. has written the first draft of this manuscript and all authors have critically read the text and contributed with inputs and revisions, and all authors read and approved the final manuscript.

## Ethics Statement

All methods were carried out following the Helsinki declaration. The study has been approved by the Ethics Committee of Tabriz University of Medical Sciences, Tabriz, Iran (Code: IR.TBZMED.REC.1400.909).

## Consent

Written informed consent was obtained from all participants.

## Conflicts of Interest

The authors declare no conflicts of interest.

## Data Availability

The datasets used and analysed during the current study available from the corresponding author on reasonable request.
